# Disruption of Midkine gene reduces traumatic brain injury through the modulation of neuroinflammation

**DOI:** 10.1186/s12974-020-1709-8

**Published:** 2020-01-29

**Authors:** Seiya Takada, Harutoshi Sakakima, Takahiro Matsuyama, Shotaro Otsuka, Kazuki Nakanishi, Kosuke Norimatsu, Yuki Itashiki, Akira Tani, Kiyoshi Kikuchi

**Affiliations:** 1 Department of Physical Therapy, School of Health Sciences, Faculty of Medicine, Kagoshima University, 8-35-1, Sakuragaoka, Kagoshima, 890-8544 Japan; 2 Department of Pulmonary Medicine, Graduate School of Medical and Dental Sciences, Kagoshima University, Kagoshima, Japan; 3 Division of Brain Science, Department of Physiology, Kurume University School of Medicine, Kurume, Japan

**Keywords:** Midkine, Traumatic brain injury, Microglia/macrophages, M1/M2 phenotype, Neuroinflammation

## Abstract

**Background:**

Midkine (MK) is a multifunctional cytokine found upregulated in the brain in the presence of different disorders characterized by neuroinflammation, including neurodegenerative disorders and ischemia. The neuroinflammatory response to traumatic brain injury (TBI) represents a key secondary injury factor that can result in further neuronal injury. In the present study, we investigated the role of endogenous MK in secondary injury, including neuroinflammation, immune response, and neuronal apoptosis activity, after TBI.

**Methods:**

Wild type (Mdk^+/+^) and MK gene deficient (Mdk^−/−^) mice were subjected to fluid percussion injury for TBI models and compared at 3, 7, and 14 days after TBI, in terms of the following: brain tissue loss, neurological deficits, microglia response, astrocytosis, expression of proinflammatory M1 and anti-inflammatory M2 microglia/macrophage phenotype markers, and apoptotic activity.

**Results:**

As opposed to Mdk^+/+^ mice, Mdk^−/−^ mice reported a significantly reduced area of brain tissue loss and an improvement in their neurological deficits. The ratios of the Iba1-immunoreactive microglia/macrophages in the perilesional site were significantly decreased in Mdk^−/−^ than in the Mdk^+/+^ mice at 3 days after TBI. However, the ratios of the glial fibrillary acidic protein immunoreactive area were similar between the two groups. The M1 phenotype marker (CD16/32) immunoreactive areas were significantly reduced in Mdk^−/−^ than in the Mdk^+/+^ mice. Likewise, the mRNA levels of the M1 phenotype markers (TNF-α, CD11b) were significantly decreased in Mdk^−/−^ mice than in Mdk^+/+^ mice. Furthermore, flow cytometry analysis identified the M2 markers, i.e., CD163^+^ macrophages cells and arginase-1^+^ microglia cells, to be significantly higher in Mdk^−/−^ than in Mdk^+/+^ mice. Finally, the ratios of apoptotic neurons were significantly decreased in the area surrounding the lesion in Mdk^−/−^ than in Mdk^+/+^ mice following TBI.

**Conclusion:**

Our findings suggest that MK-deficiency reduced tissue infiltration of microglia/macrophages and altered their polarization status thereby reducing neuroinflammation, neuronal apoptosis, and tissue loss and improving neurological outcomes after TBI. Therefore, targeting MK to modulate neuroinflammation may represent a potential therapeutic strategy for TBI management.

## Background

Traumatic brain injury (TBI) is one of the leading causes of death and disability nowadays. Indeed, more than half of trauma-related deaths can be attributed to TBI, and approximately 40% of TBI survivors in the USA develop a long-term disability [1, 2]. Its intricate pathophysiological process involves traumatic mechanical forces, which result in either primary or secondary brain injury in endothelium or the brain parenchyma [3, 4]. Secondary injury follows the primary injury-mediated neuroinflammation, immune response, oxidative stress, mitochondrial dysfunction, and apoptosis and may last from hours to days after the initial insults [5]. Furthermore, secondary injury hampers the extant neuroprotection and neurorepair mechanisms, leading to delayed and limited neurobehavioral recovery after TBI [6]. Typically, inflammation (i.e., the activation of resident microglia) is believed to play an important role in these secondary changes [7, 8].

Microglia cells are the primary mediators of the innate immune response in the central nervous system (CNS). Resident microglia cells are rapidly mobilized to the site of injury and can be polarized into either a detrimental (M1) or a beneficial (M2) phenotype depending on their host tissue microenvironments [9, 10]. M1- and M2-like microglia release several factors responsible for the modulation of secondary injury and recovery after TBI, including proinflammatory and anti-inflammatory cytokines, chemokines, nitric oxides, prostaglandins, growth factors, and superoxide species [7, 8, 11]. M1- and M2-like microglia/macrophages work in concert to fine-tune the inflammatory responses, scavenge debris, and promote the remodeling and repair following TBI [8]. Notably, the proinflammatory cytokines released by M1-like microglia, such as tumor necrosis factor-α (TNF-α) and interleukin-1β (IL-1β), are believed to contribute to neuronal cell damage [8, 12, 13]. Indeed, inhibiting microglia activation-mediated inflammation, through the regulation of both the nuclear factor-kappa B (NF-kB) and the mitogen-activated protein kinase (MAPK) signal pathway, was suggested to improve the neurobehavioral function after TBI [14]. In addition, acute administration of atorvastatin, anti-inflammatory, and immunomodulatory agents was found to attenuate the M1 microglia/macrophages phenotype and simultaneously increase the M2 phenotype post-TBI [5]. Therefore, the inhibition of microglia/macrophages activation could be a valuable therapeutic option for improving recovery in TBI patients.

Midkine (MK) is a retinoic acid-inducible heparin-binding growth factor that is transiently expressed in the mid-gestational period of mouse embryogenesis and is downregulated to inconsiderable levels in healthy adults [15]. However, MK remains upregulated during tissue repair and in several pathological conditions, including malignancies and inflammatory diseases [16]. For example, MK is expressed in the senile plaques detected in the brain of Alzheimer’s disease patients [17], in the areas surrounding cerebral infarcts [18], in injured spinal cords of rats [19], and in various human neoplasms [20]. MK promotes migration, proliferation, survival, growth, reproduction and repair, angiogenesis, and gene expression of target cells [15]. Furthermore, MK facilitates the migration of macrophages and neutrophils in various peripheral tissues, including kidney, vascular organs, and liver [21–24]. Recent studies have demonstrated that MK is involved in both the onset and progression of autoimmune diseases such as multiple sclerosis and rheumatoid arthritis [25–27]. Similarly, MK aggravates experimental autoimmune encephalomyelitis (EAE) by suppressing the development of tolerogenic dendritic cells (DCregs), thereby impairing the differentiation of regulatory T cells (Treg) [28]. Therefore, MK inhibition using RNA aptamers may provide an effective therapeutic strategy against autoimmune diseases [26]. With regard to the CNS, MK was found to modulate neuroinflammation in both the cortex and the striatum as a result of lipopolysaccharide (LPS) and amphetamine injections [29, 30]. However, the role of MK in TBI-induced neuroinflammation is yet to be elucidated. Considering that MK may regulate TBI-induced neuroinflammation, it represents a potential therapeutic option for TBI management in patients. Our previous studies demonstrated the essential role of endogenous MK in the growth, survival, and migration of various cells using wild type (Mdk^+/+^) and MK gene-deficient (Mdk^−/−^) mice [31, 32]. Here, Mdk^+/+^ and Mdk^−/−^ mouse models were utilized to investigate the role of endogenous MK in secondary injury following TBI, which includes neuroinflammation, immune response, M1 and M2 microglia/macrophage phenotype polarization, and apoptosis activity.

## Methods

### Animals

In the present study, a total of 64 Mdk^+/+^ and Mdk^−/−^ mice C57BL/6 mice (8–32 week of age; 22.3 ± 2.4 g of weight) were generated as previously described [31, 32]. In an attempt to reduce the number of animal sacrificed in the current research, both male and female mice were used. Mice were bred with a 12-h light/dark cycle and maintained at a controlled temperature (22.0 ± 1.0 °C), with free access to food and water. The experimental protocol was approved by the ethics board of the Institute of Experimental Animal Science of Kagoshima University.

### Fluid percussion injury model for TBI

Fluid percussion injury (FPI) was adopted for TBI preparation. Briefly, mice were anesthetized with 1.5–2.0% isoflurane (Pfizer Inc., Tokyo, Japan) using the MK-A110 small animal anesthetizer (Muromachi kikai, Tokyo, Japan). Mice were fixed in a stereotaxic frame, and the hair on their head was removed and sterilized with 70% ethanol. Their skull was then exposed through a midline scalp incision, and craniotomy was performed over the right parietal bone. In addition, a circular window (3.0 mm of diameter) was drilled using a dental drill to prevent damage to the dura. The surgical site was selected at 3.0 mm posterior and 3.0 mm on the right of the Bregma. Moreover, a male luer lock (3.0 mm, Isis, Osaka, Japan) filled with saline was firmly fixed on the surgical site using dental cement (Provista, Morita, Osaka, Japan). Successively, a tube of the FPI device (Dragonfly R & D INC. Model HPD-1700, USA) was tightly attached to a three-way stopcock that connected the male luer lock hub. Therefore, mice were subjected to experimental FPI, at a water pressure impact of 2.0–3.0 atm. After injury, we confirmed hemorrhage and brain edema in the circular window of the skull. Mice that present cessation of breathing or appendicular convulsions were included in this study as TBI models [33]. Finally, mice were left to recover in their cages with sufficient food and water. The weight of each mouse was periodically recorded after TBI.

### Histological and immunohistochemical analyses

**Fig. 1:**
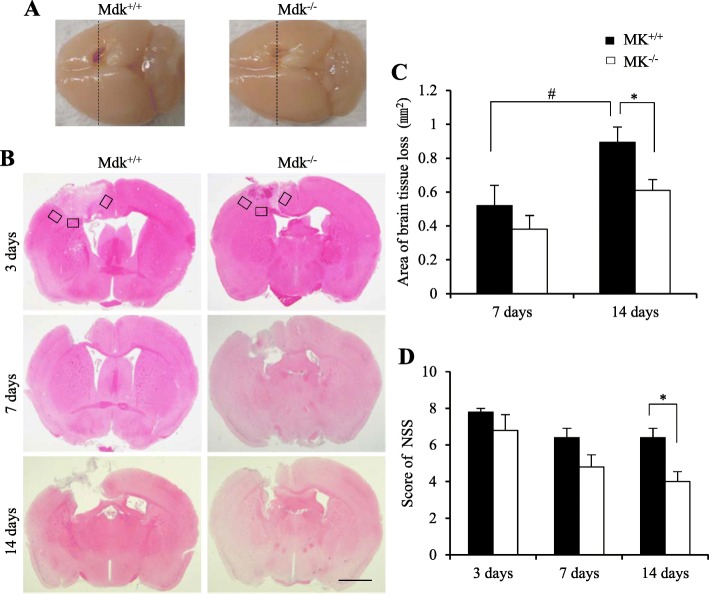
Effect of MK-deficiency on brain tissue loss and recovery of neurological deficits. Tissue loss after TBI detected using coronal hematoxylin and eosin (HE) staining, including a section obtained from the center (dashed line) of the injury site and the macro brain photographed at 14 days (**a**). TBI led to an obvious tissue loss at 7 and 14 days. Three rectangular areas in all mice were used for the quantitative analysis of the immunolabeled areas at each time-point (**b**). Mdk^−/−^ mice presented a significantly reduced tissue loss than the Mdk^+/+^ mice at 14 days after TBI (**c**). The neurological deficits in all Mdk^−/−^ mice were significantly ameliorated at 14 days after TBI (**d**). Data are presented as mean ± SE (*n* = 5 mice/group). *p < 0.05 (comparison with MK^+/+^ and Mdk^−/−^). #*p* < 0.05 (comparison with 7 days and 14 days). Scale bar = 1 mm (all panels). TBI, traumatic brain injury

Mice were sacrificed at 3, 7, and 14 days after injury for histological and immunohistochemical analyses (*n* = 5/group at each time-point). Deeply anesthetized with sodium pentobarbital, mice were perfused through the heart with physiological saline, followed by 4% paraformaldehyde in a 0.1 M phosphate buffer (pH 7.4). Subsequently, their brain was carefully removed and cut into two tissue blocks (2-mm anterior to 2-mm posterior to the injury site) using a brain slicer. The sections were then immersed in 4% paraformaldehyde in a 0.1 M phosphate buffer (pH 7.4) at 4 °C overnight. After fixation, the tissue was processed for histology and immunohistochemistry analyses. The paraffin-embedded coronal brain sections (4 μm thick) were stained with hematoxylin and eosin (HE); the HE sections of the center of injury site (Fig. 1a) were imaged at × 4 magnification using a microscope (Olympus Corp, DP21, Tokyo, Japan). The brain tissue loss at 7 and 14 days was analyzed using the Scion Image Software 4.0.3 (Scion Corp, Frederick, MD, USA), considering that the brain tissue was completely missing after TBI.

The coronal sections were immunostained with the following antibodies: rabbit anti-glial fibrillary acidic protein (GFAP; a marker of activated astrocytes) (Cosmo Bio Co., Japan; RO1003), rabbit anti-ionized calcium-binding adaptor molecule1 (Iba1; a marker of resting microglia/macrophage) (Wako, Osaka, Japan; 019-19741), rat anti-CD16/32 (a marker of M1 microglia/macrophage) (BD Bioscience, US; #553142), rabbit anti-arginase-1 (a marker of M2 microglia/macrophage) (Cell Signaling Technology, Inc., US; #93668), rabbit anti-activated caspase-3 (a marker of apoptotic activity) (Proteintech Group, Inc.; USA; 19677-1-AP), and mouse anti-neuronal nuclei (NeuN; a marker of neuron) (Abcam plc, Cambridge, UK; ab104224).

Following deparaffinization and rehydration, the endogenous peroxidase was blocked with methanol containing 3.0% hydrogen peroxide for 10 min. The sections were then rinsed three times (5 min each) with phosphate-buffered saline (PBS, pH 7.6) and blocked with 10% skimmed milk in PBS for 20 min. Successively, all sections were individually incubated at 4 °C overnight with the following antibodies: rabbit anti-GFAP (1:3000), rabbit anti-Iba1 (1:1000), rat anti-CD16/32 (1:500), rabbit anti-arginase-1 (1:1000), and rabbit anti-caspase-3 (1:200). The sections were then washed in PBS three times for 5 min each and incubated for 60 min with goat anti-rabbit IgG conjugated to either a peroxidase-labeled dextran polymer (EnVision; Dako, CA, USA) or biotinylated anti-rat IgG (1:200) and then stained with ABC (ABC Kit: Vector Laboratories, Burlingame, CA, USA), according to the manufacturer’s instructions. Finally, the sections were rinsed with PBS and their immunoreactivity was visualized through diaminobenzidine staining.

The co-localization of the rabbit anti-caspase-3 (1: 200) and the mouse anti-NeuN (1: 500) immunoreactivities were examined through immunofluorescence staining. After incubation with two primary antibodies and PBS wash, the sections were incubated for 60 min with both the FITC-conjugated goat anti-rabbit IgG (1: 200) and Alexa Fluor 555-conjugated goat anti-mouse IgG antibodies (1: 200). The sections were washed with PBS and counterstained with 4′, 6-diamino-2-phenylindole for 10 min. Finally, they were mounted with an aqueous mounting media and immunofluorescent staining was observed with a fluorescence microscope (EVOS f1; AMG, Mill Creek, WA, USA).

### Evaluation of neurological deficits

Mice (*n* = 5/group) were scored neurologically for focal deficits using a 28-point neurological severity score (NSS) [34]. Briefly, this system included comprehensive assessment of motor, sensory, reflex, and balance. The highest test score possible was 28 points, representing the most serious neurological impairment. Prior to TBI, all mice scored zero. Following TBI, the NSS was administered to mice at 3, 7, and 14 days, and the scoring was performed by two individuals.

### Quantitative analysis of immunolabeled areas

Three areas surrounding the lesion in each immunostained section were imaged at × 40 magnification using a microscope and a camera without visual field overlap (Fig. 1a). The ratios of GFAP-, Iba1-, CD16/32-, ariginase-1-, and caspase-3-positive cells were quantitatively measured between the two groups in coronal sections using the Scion Image software 4.0.3 at 3, 7, and 14 days after TBI. Moreover, at 7 days after TBI, sections obtained from two areas in the motor cortex of the surrounding lesion, with a co-localization of caspase-3 and NeuN were imaged (× 20 magnification) following immunofluorencent staining. Furthermore, the ratios of caspase-3- and NeuN-positive neurons were counted in the surrounding lesion (0.21 mm^2^) and a quantitative analysis of each immunolabeled area was performed by two to three individuals.

### Reverse transcriptase quantitative PCR (RT-qPCR)

All mice were euthanized through deep anesthesia with sodium pentobarbital 3 days after TBI (*n* = 3–4/group). Therefore, the total RNA was isolated from the injured brain (100 mg) using the Trizol reagent (Cosmo Bio Co., Japan, 5631) and the cDNA was synthesized using the Verso cDNA Kit (Thermo Fisher Scientific, Massachusetts, USA) according to the manufacturer’s instructions. This was then amplified by reverse transcriptase PCR using the SYBR green PCR Master Mix (Applied Biosystem; Thermo Fisher Scientific, Inc.). Specifically, a two-step qPCR was performed (95 °C for 15 s, 60 °C for 30 s for 40 cycles) with specific primers. The comparative cycle threshold (Ct) analysis (ΔΔCt) was performed to evaluate the fold-change in mRNA expression using GAPDH expression as the reference. We checked that GAPDH expression does not differ between experiment groups. The sequences of the primer pairs for the M1 phenotype genes are the following: (1) tumor necrotic factor-α (TNF-α): forward, 5′-CGTCGTAGCAAACCACCAAAG-3′ and reverse, 5′-GAGATAGCAAATCGGCTGACG-3′; (2) CD16: forward, 5′-TTTGGACACCCAGATGTTTCA-3′ and reverse, 5′-GTCTTCCTTGAGCACCTGGATC-3′; (3) CD11b: forward, 5′-CCAAGACGATCTCAGCATCA-3′ and reverse, 5′-TTCTGGCTTGCTGAATCCTT-3′. In contrast, the sequences of the primer pairs for the M2 phenotype genes are as follows: (1) arginase-1: forward, 5′-TCACCTGAGCTTTGATGTCG-3′ and reverse, 5′-CTGAAAGGAGCCCTGTCTTG-3′; (2) CD206; forward, 5′-CAAGGAAGGTTGGCATTTGT-3′ and reverse, 5′-CCTTTCAGTCCTTTGCAAGC-3′. The sequences of the primer pairs for the GAPDH genes are as follows: forward, 5′-AACTTTGGCATTGTGGAAGG-3′ and reverse, 5′-ACACATTGGGGTAGGAACA-3′.

### Flow cytometry

Deeply anesthetized with sodium pentobarbital, mice were perfused with physiological saline through the heart and their ipsilateral brain was promptly removed 3 days after TBI (*n* = 4/group). Tissue debris were then incubated via Percoll gradient centrifugation to obtain a single-cell suspension. Prior to antibody labeling, the cell suspension was incubated at 4 °C for 20 min with the anti-mouse CD16/32 FACS buffer (BD pharmingen, New Jersey, USA; Clone 2.4G2) in FACS buffer (HBSS buffer containing 2% FBS) to prevent non-specific binding of immunoglobulin to macrophage Fc receptors. Cells were lysed in the FACS buffer containing the following fluorescent labels and incubated at 4 °C for 20 min in the dark: CD4- BV421(BD pharmingen, NJ, USA; Clone GK1.5), CD3e- BV510 (Biolegend, CA, USA; Clone 145- 2C11), CD45.2- Alexa Fluor 700 (Biolegend, CA, USA; Clone 104), CD25- PECy7 (Biolegend; Clone PC61), and CD11b-FITC (TONBO bioscience, CA, USA; Clone M1/70). To differentiate M1 and M2 microglia/macrophages phenotype, we used antibodies against the iNOS-PE (eBioscience, California, USA, 12-5920-82; Clone CXNFT), arginase-1-APC (eBioscience, CA, USA, 17-3697-82; Clone A1exF5), CD80-PE (eBioscience, CA, USA, 12-0801-81; Clone 16-10A1), and CD163-APC (eBioscience, CA, USA, 17-1631-82; Clone TNKUPJ) monoclonal antibodies. All cells were then incubated at 4 °C for 20 min in the dark, and their fluorescence was analyzed with the FAC Scan analyzer (BD bioscience, CA, USA). Finally, we calculated the percentages of total microglia or macrophage cells number.

### Statistical analysis

Statistical analyses were performed with either parametric or non-parametric tests after Shapiro Wilk test. Either the independent Student *t* test or the Mann–Whitney *U* test was applied for between-group analyses. The time course for NNS was analyzed through the Friedman test. In addition, the time course for the percentages of immunostained areas was analyzed using either a one-way analysis of variance (ANOVA) or the Kruskal-Wallis test, followed by Bonferroni’s post hoc tests for multiple comparisons. A *p* value of < 0.05 was considered statistically significant. Data are expressed as mean ± standard error (SE). All data were analyzed using SPSS version 24 (IBM, Chicago, IL, USA).

## Results

### MK-deficiency alleviates brain tissue loss and neurological deficits following TBI

To assess the FPI-induced morphological changes, HE-stained sections of the lesions were evaluated at 3, 7, and 14 days after TBI (Fig. 1a, b). Substantial brain damage was seen in the cortical layers with an extension into the corpus callosum of the ipsilateral hemisphere at 3 days after injury in all mice. Considering that brain tissue loss became obvious at 7 and 14 days following TBI, we estimated the area of brain tissue loss in all mice at these two time-points. While Mdk^+/+^ mice presented a significant increase in the area of brain tissue loss over time (Fig. 1c; *p* = 0.037, *t* test), the same was not observed for Mdk^−/−^ mice. In contrast, at 14 days after TBI, Mdk^−/−^ mice showed a significantly smaller area of brain tissue loss compared to Mdk^+/+^ mice (Fig. 1c; *p* = 0.033, *t* test). Nonetheless, a significant difference between Mdk^+/+^ and the Mdk^−/−^ mice at 7 days after TBI was not observed regarding brain tissue loss.

The neurological deficits in mice were assessed using the NSS system (Fig. 1d). While they were found to be absent in both mice groups before TBI, all mice exhibited severe neurological deficits after the injury, which however improved with time. Specifically, as opposed to Mdk^+/+^ mice, the neurological deficits in Mdk^−/−^ mice were significantly ameliorated at 14 days after TBI (*p* = 0.032, Mann–Whitney *U* test).

### MK-deficiency lessened neuroinflammation through a decrease in microglia responses and in M1-like microglia/macrophages expression during the early phase after TBI

**Fig. 2:**
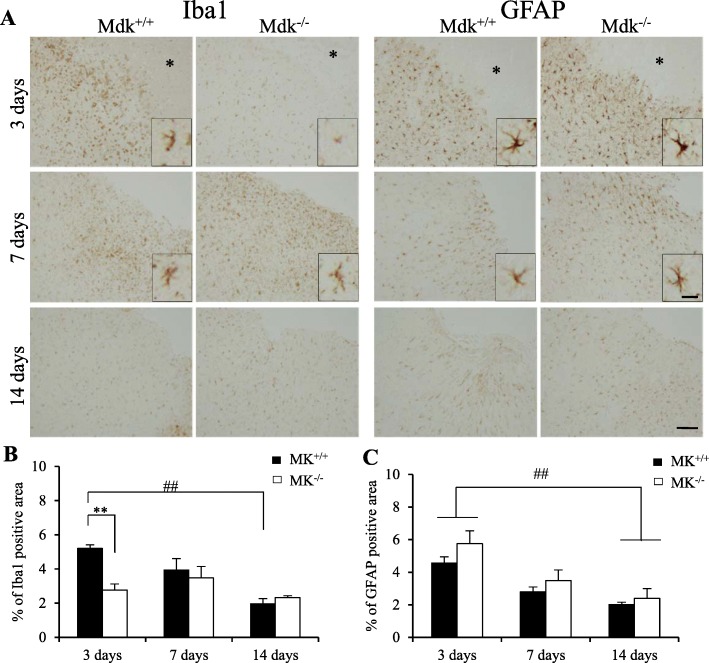
Effect of MK-deficiency on microglia response and astrocytosis after TBI. Iba1-immunoreactive microglia/macrophages surrounding the lesion site (*) was significantly decreased in Mdk^−/−^ compared to Mdk^+/+^ mice at 3 days after TBI (**a**, **b**). However, there were no difference in GFAP-immunoreactivities between MK^+/+^ and Mdk^−/−^ mice at each time-point. (**a**, **c**). Furthermore, although the density and processes of Iba1-positive microglia cells in Mdk^−/−^ mice were smaller than those of Mdk^+/+^ mice at 3 days, GFAP-positive astrocytes were not observed (**a**). Data are presented as mean ± SE (*n* = 5 mice/group). **p < 0.01 (comparison with MK^+/+^ and Mdk^−/−^). ##*p* < 0.01 (comparison with 3 days and 14 days). Scale bar = 100 μm (all panels) and 20 μm (all high magnification panels). GFAP, glia fibrillary acidic protein

To investigate the inflammatory responses to TBI, microglial or astrocytic responses were determined via immunohistochemistry (Fig. 2). We found significantly lesser Iba1-immunoreactive microglia/macrophages at the perilesional site of Mdk^−/−^ than those of Mdk^+/+^ mice at 3 days (*p* = 0.0001, Mann–Whitney *U* test). In addition, the density and processes of Iba1-positive microglia cells in Mdk^−/−^ mice were smaller than those in Mdk^+/+^ mice (Fig. 2a). Although Iba1-positive cells slightly increased at 7 days, no significant difference in the time course after TBI was observed between the groups. Moreover, Iba1-immunoreactivities in Mdk^+/+^ mice significantly decreased at 14 days than at 3 days after TBI (*p* = 0.001, one-way ANOVA) and exhibited similar degrees of Iba1-immunoreactive areas to Mdk^−/−^ mice at 14 days after TBI. Although GFAP-positive astrocyte immunoreactivity was observed at 3 days in all mice, it significantly reduced at 14 days than at 3 days after TBI (*p* < 0.05, one-way ANOVA). However, no significant difference was observed in the ratios of GFAP-positive over GFAP-negative areas between mice at any time-point after TBI (Fig. 2c). Unexpectedly, morphological changes between mice such as density and processes of GFAP-positive astrocytes were not observed (Fig. 2a).

**Fig. 3:**
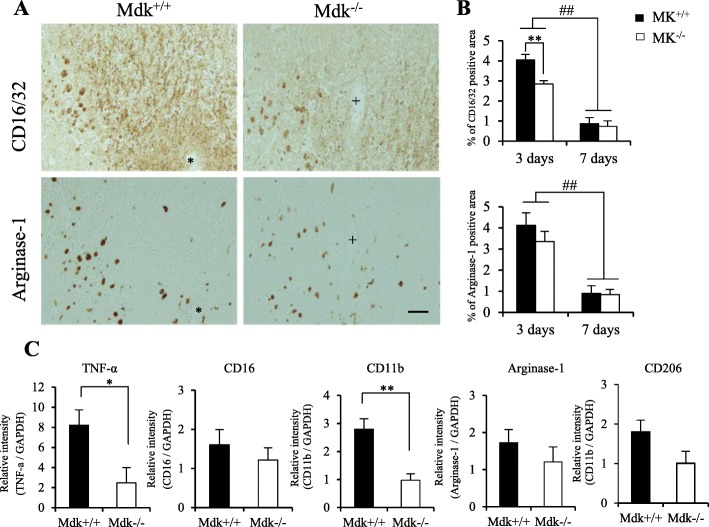
Effect of MK-deficiency on M1 and M2 microglia/macrophages phenotype marker after TBI. The M1 and M2 phenotype markers (CD16/32 and arginase-1, respectively) were expressed in the perilesional site of Mdk^+/+^ and Mdk^−/−^ mice at 3 days (**a**). The immunohistochemical staining was performed through serial sections of mice (corresponding to * and + in **a**). The ratios of the CD16/32-immunoreactive area were significantly reduced in Mdk^−/−^ mice compared to Mdk^+/+^ mice at 3 days. The CD16/32- and arginase-1-immunoreactive areas were significantly decreased at 7 days (**b**). RT-qPCR analysis revealed the mRNA levels of the M1 phenotype markers (TNF-α, CD11b) to be significantly downregulated in Mdk^−/−^than in Mdk^+/+^ mice (**c**). Data are presented as mean ± SE (*n* = 5 mice/group in immunohistochemistry, *n* = 3–4 mice/group in RT-qPCR). *p < 0.05, **p < 0.01 (comparison with MK^+/+^ and Mdk^−/−^). ##*p* < 0.01 (comparison with 3 days and 7 days). Scale bar = 50 μm (all panels)

Successively, considering that Iba1-positive microglia/macrophages cells were significantly decreased in acute phase after TBI in Mdk^−/−^ mice, the expression of microglia/macrophages was determined through immunohistochemistry using M1 and M2 phenotypes markers at 3 and 7 days (Fig. 3a, b). The M1 and M2 phenotype markers (CD16/32 and arginase-1, respectively) were expressed in the perilesional site in all mice at 3 days; however, they significantly decreased at 7 days after TBI (Fig. 3a, b; *p* < 0.01, *t* test). Although the ratios of the CD16/32-immunoreactive area were significantly reduced in Mdk^−/−^ than in Mdk^+/+^ mice at 3 days (Fig. 3b; *p* = 0.006, *t* test,), a significant difference between mice at 7 days after TBI was not observed. Similarly, at each time-point after TBI, the ratios of the arginase-1-immunoreactive area were not significantly different between mice at any time of analysis (Fig. 3b).

### MK-deficiency altered the polarization of M1 and M2 microglia/macrophages phenotype markers

Polarized microglia/macrophages can be distinguished by surface marker expression and cytokine/chemokine production. Therefore, we examined the markers and the functional cytokine mRNA levels of the M1 (TNF-α, CD16, CD11b) and M2 (arginase-1, CD206) phenotypes by RT-qPCR analysis at 3 days (Fig. 3c). The mRNA levels of the M1 phenotype markers (TNF-α, CD11b) were significantly lower in Mdk^−/−^ than in Mdk^+/+^ mice (*p* < 0.05, *t* test). In contrast, the mRNA levels of the M2 phenotype markers were similar between mice.

**Fig. 4:**
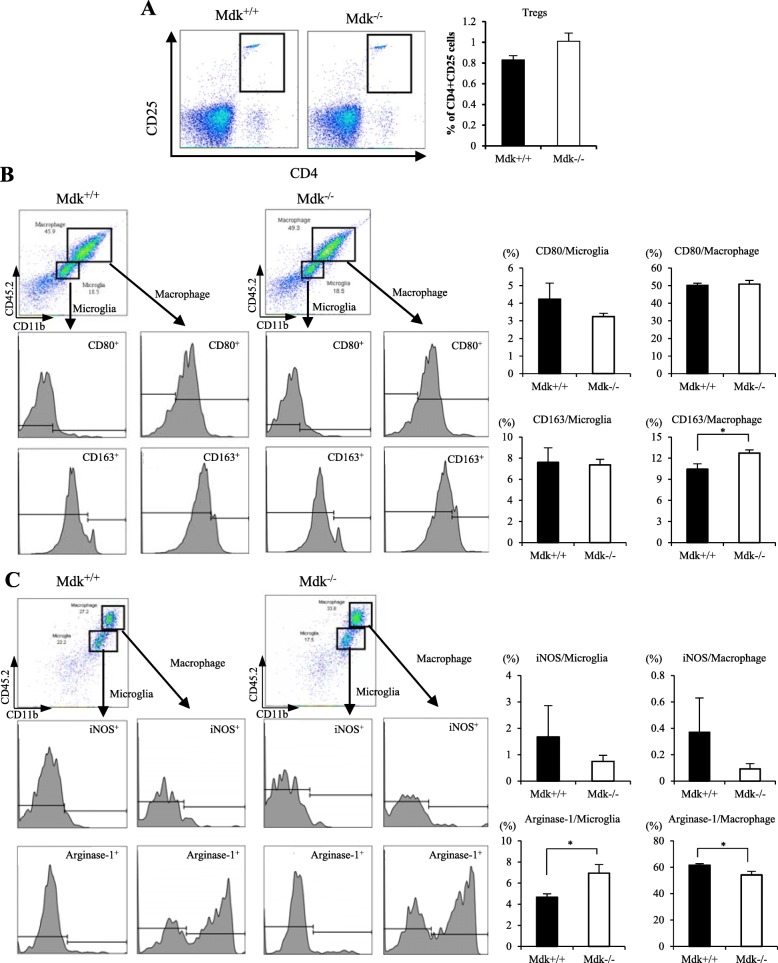
Effect of MK-deficiency on the Treg population and the M1 and M2 microglia/macrophages phenotype markers after TBI. The expression of CD4^+^CD25^+^ T cells (Tregs) was higher in Mdk^−/−^ than in the Mdk^+/+^ mice, although a significant difference between two groups was not observed (**a**). The expressions of the M2 phenotype markers, i.e., CD163^+^ macrophages and arginase-1^+^ microglia, were significantly higher in Mdk^−/−^ mice than in the Mdk^+/+^ mice (**b**, **c**). In contrast, the expression of arginase-1^+^ macrophages was significantly lower in Mdk^−/−^ than Mdk^+/+^ mice (**c**). Data are presented as mean ± SE (*n* = 4 mice/group). *p < 0.05 (comparison with MK^+/+^ and Mdk^−/−^)

In addition, cells isolated from the brain lesions were analyzed for CD4^+^CD25^+^ T cell (Treg), M1 phenotype markers (CD80^+^, iNOS^+^), and M2 phenotype markers (CD163^+^, arginase-1^+^) through flow cytometry analysis at 3 days after TBI (Fig. 4). We found the expression of CD4^+^CD25^+^ T cells to be higher in Mdk^−/−^ than in Mdk^+/+^ mice. However, a statistically difference was not observed (Fig. 4a; *p* = 0.090, *t* test). The expression of the M1 markers, i.e., CD80^+^ microglia and iNOS^+^ microglia/macrophages, were decreased in Mdk^−/−^ than in the Mdk^+/+^ mice. However, a significant difference was not observed. In contrast, the expression of the M2 markers, i.e., CD163^+^ macrophages and arginase-1^+^ microglia, was significantly higher in Mdk^−/−^ compared to Mdk^+/+^ mice (Fig. 4b, c; *p* < 0.05, *t* test). Furthermore, arginase-1^+^ macrophage expression was significantly lower in Mdk^−/−^ than in the Mdk^+/+^ mice (Fig. 4b; *p* < 0.05, *t* test).

### MK-deficiency reduced neuronal apoptotic cell death surrounding lesions after TBI

**Fig. 5:**
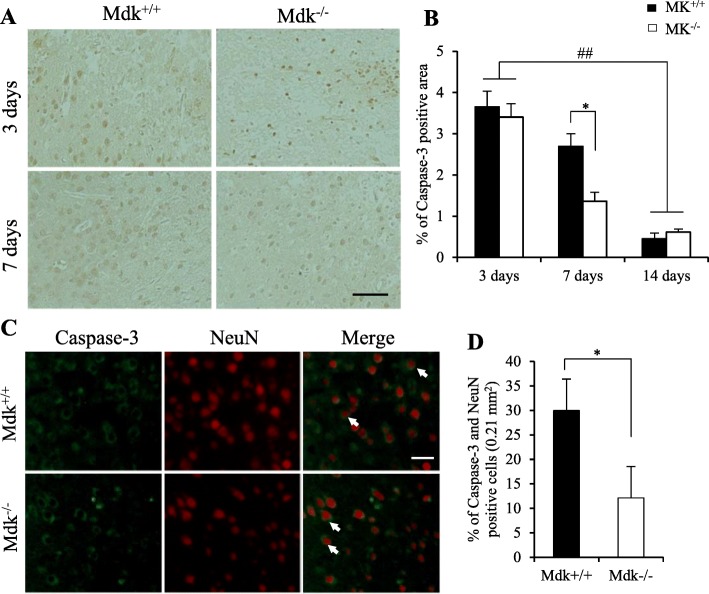
Effect of MK-deficiency on neuronal apoptosis activity after TBI. Caspase-3-positive cells were detected in the lesion and perilesional areas (**a**). A higher expression of the caspase-3-positive area was seen in Mdk^−/−^ than in the Mdk^+/+^ mice at 7 days after TBI (**b**). Moreover, caspase-3-immuno-positive cells co-localized with a neuronal marker (NeuN), indicating neuronal apoptosis surrounding the lesion at 7 days after TBI (**c**). The populations of apoptotic neurons (arrow) were significantly lower in Mdk^−/−^ than in Mdk^+/+^ mice (**d**). Data are presented as mean ± SE (*n* = 5 mice/group). *p < 0.05 (comparison with MK^+/+^ and Mdk^−/−^). ##*p* < 0.01 (comparison with 3 days and 14 days). Scale bar = 50 μm (all panels of **a**) and 25 μm (all panels of **c**)

Finally, the immunoreactivity of the activated apoptotic marker caspase-3 in the perilesional site after TBI was assessed (Fig. 5). Caspase-3-positive cells were detected in the lesion and the perilesional area at 3 and 7 days post-TBI. However, their expression decreased over time and they were scarcely detected at 14 days after TBI (Fig. 5a, b). Similarly, their expression was significantly lower in Mdk^−/−^ than in Mdk^+/+^ mice at 7 days following TBI (*p* = 0.008, *t* test). Immunofluorescence staining showed caspase-3-immuno-positive cells to co-localize with the neuronal marker (NeuN), suggesting neuronal apoptosis surrounding the lesion at 7 days after TBI (Fig. 5c). Therefore, we investigated the ratios of caspase-3- and NeuN-positive neurons surrounding the lesion area in all mice and found the ratios of apoptotic neurons to be significantly lower in Mdk^−/−^ than in Mdk^+/+^ mice at 7 days following TBI (Fig. 5d; *p* = 0.038, *t* test).

## Discussion

This study showed MK-deficiency to both reduce the lesion volume and improve the neurological deficits after TBI through the modulation of both neuroinflammation and neuronal apoptosis. Indeed, this results in a decrease in activation of microglia/macrophages, proinflammatory cytokine, and caspase-3 activity surrounding the lesion at the early phase after TBI. In addition, MK-deficiency was found to suppress M1 microglia/macrophages phenotype marker and to promote M2 phenotype. MK acts as a proinflammatory cytokine and contributes to chronic inflammation through the promotion of chemotaxis and tissue infiltration of neutrophils and microglia/macrophages [27]. However, MK administration may not directly activate astrocyte or microglia in vitro [35]. Thus, the reduced microglia activation in Mdk^−/−^ mice may have resulted owing to an interference to the migration of neutrophils and macrophages to the injury site at the acute phase after TBI. Wang et al. [26] demonstrated that Mdk^−/−^ EAE mice had fewer infiltrating inflammatory cells in the lumbar spinal cord than Mdk^+/+^ EAE mice. In addition, overexpression of the MK gene mediated by p53 remodels the immunosuppressive microenvironment of a glioblastoma by promoting the M2 polarization of microglia [36]. Collectively, our findings suggest the lack of expression of MK gene to reduce both chemotaxis and tissue infiltration of microglia/macrophages, as well as to alter the microglia/macrophages polarization status. This ameliorates neuroinflammation and neuronal apoptosis, consequently leading to reduced tissue loss and favorable neurological outcomes after TBI.

Microglia/macrophages are the primary mediators of the innate response to injury [11]. In fact, a large number of microglia and macrophages proliferate and infiltrate the injury site after TBI [9]. Experimental studies indicate that while neutrophil infiltration in the TBI brain is maximal at 1 day post-injury, it is followed by accumulation of leukocyte subsets that peak at about 3 days post-injury [37]. Inflammatory monocytes are preferentially recruited to brain area subjected to TBI, and they predominate the lesion site at 3 days [8]. In both the spinal cord and the ischemic brain injury, a majority of the microglia and recruited macrophages at the injury site have mixed M1- and M2-like activation profiles [38, 39]. In fact, the mRNA expression of the M1 and M2 phenotype markers rapidly increases after TBI or spinal cord injury [7, 38]. Although the M2 gene expression may fluctuate, the increase in M1 gene expression is sustained over time after TBI [7]. In concordance to previous studies, we also observed a mixed pattern of M1 and M2 phenotype marker expression using immunohistochemistry, RT-qPCR, and flow cytometry. Specially, our results indicated that Mdk^−/−^ mice presented reduced activations of microglia/macrophages in the perilesional site, as well as decreased mRNA levels of M1 phenotype markers (TNF-α, CD11b). In addition, they also showed increased M2 phenotype marker cells (CD163, arginase-1) in the acute phase after TBI. While TNF-α is an important glia-mediated proinflammatory cytokine participating in the caspase-3-dependent cell death [40], arginase-1 may be most relevant for CNS repair [38]. Our findings suggest that absence of the MK gene might modulate the M1 and M2 phenotype polarization of microglia/macrophages, thereby alleviating neuroinflammation and neuronal apoptosis after TBI. However, the expression of the M2 phenotype marker was not significantly enhanced, as seen through the immunohistochemical and RT-qPCR analyses. In contrast, the expression of the M1 phenotype marker did not show a significant reduction, as determined by flow cytometry. These discordances may depend on the detection method and the phenotype marker (e.g., surface marker expression and cytokine/chemokine production) used. In addition, the Treg population in TBI brains was not found to be significantly different between Mdk^+/+^ and Mdk^−/−^ mice. Tregs were reported to both inhibit microglia/macrophages activation and shift their polarization towards the protective M2 phenotype although CD4^+^CD25^−^ T cells displayed the opposite effects by promoting M1 polarization [41–43]. Considering that MK aggravates EAE by decreasing regulatory CD4^+^CD25^+^Foxp3^+^ T cells (Tregs) [26, 28], further studies should be conducted to clarify the mechanism underlying MK-induced microglia/macrophages polarization after TBI. To the best of our knowledge, this study was the first to suggest that MK could regulate microglia/macrophages polarization and neuroinflammation following experimental TBI.

MK may act as a modulator of CNS neuroinflammation depending on the inflammatory stimulus [29, 44]. Although our results showed MK-deficiency to regulate microglia-mediated neuroinflammation, the same was not valid for astrocytosis in the perilesional cortex after TBI. While MK reduces the microglia responses and astrocytosis induced by amphetamine in the striatum, LPS-induced neuroinflammation seems to be potentiated by MK [44]. Therefore, TBI-induced glial response may be different from the drug-induced one. In addition, MK-induced microglia response and astrocytosis may present different regulation patterns, based on either the inflammatory stimulus or the observation site (e.g., the cortex or striatum).

In mammals, MK and pleiotrophin (PTN) present an approximately 50% similarity in their amino acid sequence and comprise a two-member family of heparin-binding cytokines [16]. Both cytokines are novel modulators of neuroinflammation and play different roles based on the inflammatory stimulus and the duration of the neuroinflammatory processes. Therefore, the MK/PTN signaling pathways are novel therapeutic strategies to modulate neuroinflammation in acute and chronic pathological states [44]. Inhibiting MK as well as PTN may be beneficial in neuroinflammatory states that promote exacerbation of neurodegeneration. The potential inhibition of both MK and PTN has been previously studied for the treatment of malignant disease models and EAE mice, including siRNA, shRNA antibody, and RNA aptamers [26, 45, 46]. In contrast, intrathecal administration of MK showed functional recovery after rat model of spinal cord injury [35]. Collectively, MK may be a multifunctional cytokine with a dual therapeutic potential for CNS injury. Therefore, further studies investigating the neuroprotective mechanisms and therapeutic potentials of MK are warranted. In the present study, MK was proposed to be an important factor and a potent regulator of neuroinflammation via the moderation of microglia/macrophages polarization after TBI. Therefore, the inhibiting action of MK may be considered as a potential therapeutic advantage to modulate neuroinflammation when treating TBI.

## Conclusions

The present study demonstrated MK-deficiency to reduce tissue infiltration of microglia/macrophages and alter the M1- and M2-like microglia/macrophages polarization status. These were indicated to result in alleviated neuroinflammation and decreased neuronal apoptosis, leading to reduced tissue loss and improved neurological outcomes after TBI. Therefore, targeting MK to modulate neuroinflammation may be a potential therapeutic strategy for TBI.

## Data Availability

The datasets used and/or analyzed during the current study are available from the corresponding author on reasonable request.

## Abbreviations

ANOVA: One-way analysis of variance
CNS: Central nervous system
EAE: Experimental autoimmune encephalomyelitis
FPI: Fluid percussion injury
GFAP: Glial fibrillary acidic protein
HE: Hematoxylin and eosin
Iba1: Ionized calcium-binding adaptor molecule1
IL-10: Interleukin-10
IL-1β: Interleukin-1β
iNOS: Inducible nitric oxide synthase
LPS: Lipopolysaccharide
MAPK: Mitogen-activated protein kinase
Mdk^−/−^: MK gene deficient
Mdk^+/+^: Wild type
MK: Midkine
NF-kB: Nuclear factor-kappa B
NSS: Neurological Severity Score
PTN: Pleiotrophin
RT-qPCR: Reverse transcriptase quantitative PCR
TBI: Traumatic brain injury
TNF-α: Tumor necrosis factor-α
Treg: Regulatory T cell
